# Body Mass Index and Mortality: A 10-Year Prospective Study in China

**DOI:** 10.1038/srep31609

**Published:** 2016-08-22

**Authors:** Jian-Bing Wang, Meng-Jia Gu, Peng Shen, Qiu-Chi Huang, Chen-Zheng Bao, Zhen-Hua Ye, You-Qing Wang, Mamat Mayila, Ding Ye, Shi-Tong Gu, Hong-Bo Lin, Kun Chen

**Affiliations:** 1 Department of Epidemiology and Biostatistics, School of Public Health, Zhejiang University, Hangzhou 310058, China; 2 Research Center for Air Pollution and Health, Zhejiang University, Hangzhou 310058, China; 3 Yinzhou District Center for Disease Control and Prevention, Ningbo, 315100, China; 4 Zhejiang Cancer Hospital, 310022, China; 5 Michigan State University, 48824, USA

## Abstract

Although several studies have evaluated the role of body weight as a risk factor for mortality, most studies have been conducted in Western populations and the findings remain controversial. We performed a prospective study to examine the association between body mass index (BMI) and all-cause mortality in Yinzhou District, Ningbo, China. At baseline, 384,533 subjects were recruited through the Yinzhou Health Information System between 2004 and 2009. The final analysis was restricted to 372,793 participants (178,333 men and 194,460 women) aged 18 years and older. Cox proportional hazards models were used to estimate hazard ratios(HRs) and 95% confidence intervals(CIs). We found an increased risk of all-cause mortality among individuals with BMI levels <22.5–24.9, although several groups were not statistically significant—adjusted HRs for persons with BMIs of <15.0, 15.0–17.4, 17.5–19.9, and 20.0–22.4 were 1.61(95% CI: 1.17–2.23), 1.07(0.94–1.20), 1.04(0.98–1.10), 1.06(1.02–1.11), respectively. In the upper BMI range, subjects with BMIs of 25.0–34.9 had a reduced risk of all-cause mortality. Sensitivity analyses excluding smokers, those with prevalent chronic disease or those with less than four years of follow-up did not materially alter these results. Our findings provide evidence for an inverse association of BMI and mortality in this population.

Being overweight or obses are defined as body mass index (BMI) of 25–30 or more than 30 kg/m^2^, respectively^1^. The prevalence of obesity is increasing to epidemic proportions at an alarming rate worldwide. Epidemiologic evidence has indicated that about 1.9 billion adults worldwide are overweight and over 600 million adults are obese^2^. The prevalence rates of overweight and obesity have also increased greatly over the past decade in China^3^. Observational studies have demonstrated the association of obesity with several chronic diseases including cancer, cardiovascular disease, and diabetes^4^,^5^,^6^. The association of body weight and mortality has been of interest to epidemiologists and health professionals for several decades. However, the relationship between overweight and all-cause mortality remains controversial. A number of studies have indicated a lower risk for overweight, while others have suggested either no risk or an increased risk. Reports from the Cancer Prevention Study II (CPS II)^7^, found that overweight was associated with excess mortality. However, a recent meta-analysis conducted by Flegal *et al*.^8^, which included 97 studies with a combined sample size of 2.88 million and more than 270,000 deaths, found that overweight was associated with a decreased risk of mortality relative to normal weight (hazard ratio (HR) = 0.94, 95% confidence interval (CI): 0.91–0.96). The discrepancy in results may reflect the differences in age distribution, BMI distribution, reference BMI category used, preexisting chronic disease, and potential confounding factors.

Most of these previous studies have been conducted in Western populations in which a high proportion of participants are obese. It is still unclear whether the findings of these studies can be extrapolated to Asia populations.

Here, we report the results from a prospective study of 384,533 subjects in China based on data from Yinzhou Health Information System (YHIS), a regional health information system covering all individuals in the Yinzhou District of Ningbo, Zhejiang Province. In total there were 12,843 deaths over ten years of follow-up. The purpose of our study was to examine the association between BMI and the risk of all-cause mortality in the YHIS study population.

## Results

Of the 372,793 people in the YHIS study population, more than half (52%) were women. The mean age at baseline was 46 years, and the average BMI was 22.8 kg/m^2^ for both sexes. Most participants (81.0%) had a normal BMI (18.5 to <25 kg/m^2^); 3.0% had a low BMI (<18.5 kg/m^2^) and 16.0% a high BMI (≥25 kg/m^2^). Higher education (≥6 years) was more common among participants with low BMI. Cigarette smoking, alcohol consumption, and physical inactivity were more prevalent among participants with high BMI (Table 1). During a mean follow-up period of 8 years, 6,678 men died (including 2,743 from cancer, 1,704 from cardiovascular disease, 936 from respiratory disease, 147 from diabetes, 131 from gastrointestinal disease and 937 from other causes) and 6,165 women died (including 1,616 from cancer, 2,078 from cardiovascular disease, 1,001 from respiratory disease, 267 from diabetes, 119 from gastrointestinal disease and 1,031 from other causes).

The absolute risk of death varied substantially among BMI subgroups. The age-standardized rates of death from all causes for 10 categories of BMI are displayed in Table 2), and show an approximately linear inverse association between BMI and all cause mortality (*P* < 0.05).

Table 3 presents hazard ratios between BMI and all-cause mortality, and shows a similar nearly linear inverse association. The directionality of the crude associations with BMI subgroups remained after multivariate adjustment for potential confounding factors, including age, sex, cigarette smoking, alcohol drinking, marital status, education and physical activity. As compared with subjects with a BMI of 22.5–24.9, individuals with a BMI of less than 15.0 had an adjusted HR of 1.61(95% CI: 1.17–2.23) for all-cause mortality, and the adjusted HRs for individuals with BMIs of 15.0 to 17.4, 17.5 to 19.9, and 20.0 to 22.4 were 1.07(95% CI: 0.94–1.20), 1.04(95% CI: 0.98–1.10), and 1.06(95% CI: 1.02–1.11), respectively. Participants with BMIs of 25.0–34.9 had a decreased risk of all-cause mortality. These associations remained consistent in men and women, but were a bit stronger among men. The associations were also slightly stronger among younger participants (<60 years) than among older participants (≥60 years), although this difference was not statistically significant. When we used the WHO/National Heart, Lung, and Blood Institute criteria, with a BMI of 18.5–24.9 as a reference, the HRs for all-cause mortality were 1.03(95% CI: 0.95–1.10) for BMI < 18.5, 0.69(0.65–0.73) for BMI of 25.0–29.9, and 0.52(0.42–0.64) for BMI ≥ 30.0.

**Figure 1:**
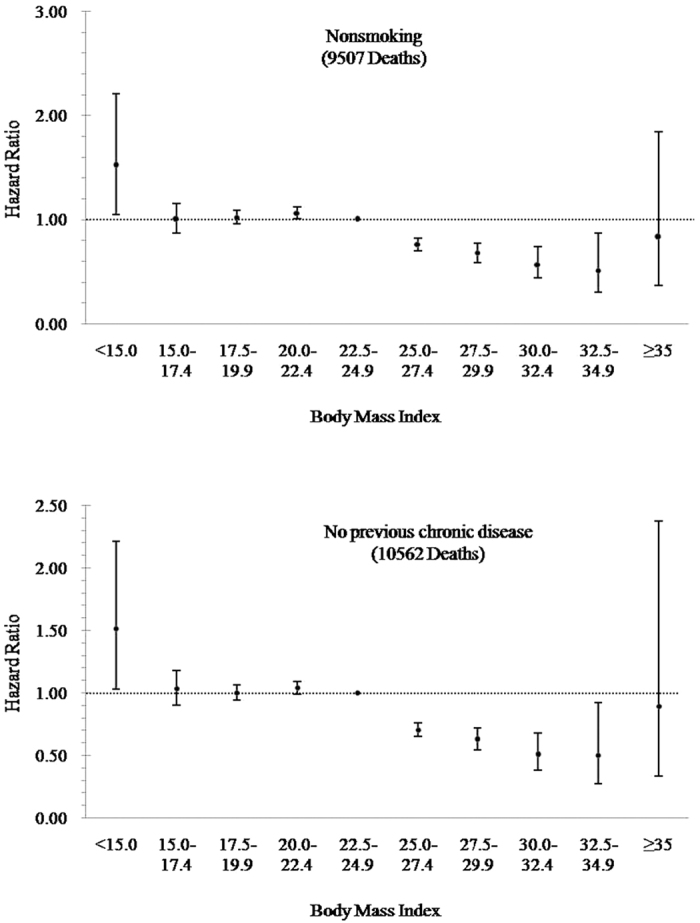
Hazard ratios for death from all-cause according to Body Mass Index among nonsmokers and participants with no previous chronic disease. Sensitivity analyses were conducted by excluding participants who smoked or had preexisting chronic disease. The hazard ratios were adjusted for age, sex, drinking, marital status, education, physical activity and cigarette smoking (further adjustment in the analysis for participants who had no preexisting chronic disease). The reference level was a BMI of 22.5–24.9.

**Figure 2:**
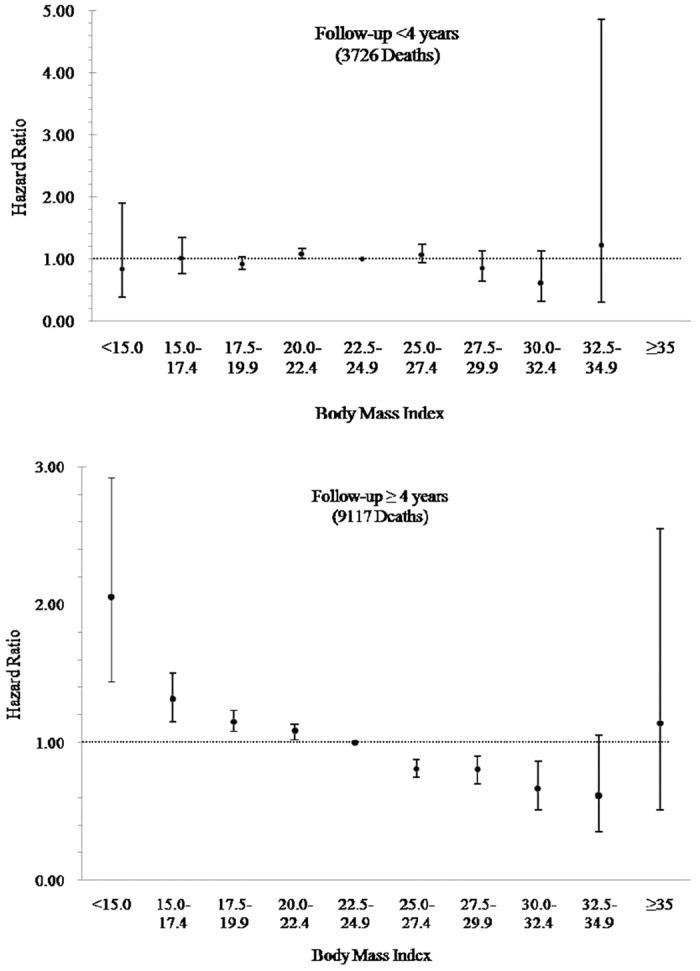
Hazard ratios for death from all-cause according to Body Mass Index by follow-up period. The hazard ratios were stratified by follow-up period (<4 years and ≥4 years), and were adjusted for age, sex, smoking, drinking, marital status, education and physical activity. The reference level was a BMI of 22.5–24.9. Hazard ratio for BMI ≥ 35 kg/m^2^ in the follow-up of <4 years period cannot be estimated due to the small cases.

When we excluded participants who smoked or had preexisting chronic disease, the associations between BMI and risk of all-cause mortality remained similar (Fig. 1). When stratifying by duration of follow-up, results appeared different across the follow-up of less than four years and ≥ four years. An elevated but nonsignificant risk of death was associated with the highest BMI category (HR = 1.14, 95% CI: 0.51–2.55) in the stratified follow-up analysis of individuals with a follow-up of four years or more (Fig. 2).

## Discussion

This study prospectively examined the association between BMI categories and risk of all-cause mortality in the population of Yinzhou District of Ningbo, Zhejiang Province, China. In this large prospective study, we found a relatively higher risk of mortality among lower BMI participants and a relatively lower risk of mortality among individuals with a higher BMI. As compared with persons who had a BMI of 22.5–24.9, participants who were underweight (BMI < 15.0) had the highest risk of mortality (HR = 1.61, 95% CI: 1.17–2.23) and participants with a BMI of 30.0–32.4 had the lowest risk (HR = 0.48, 95% CI: 0.29–0.80). Subgroup analyses by sex, age at baseline, smoking, previous chronic disease, and duration of follow-up did not alter our findings.

Our study included a high proportion of participants with a low BMI, and confirmed the findings for the association of underweight with risk of all-cause mortality. A number of epidemiological studies have revealed that among underweight persons, the increased risk was driven by respiratory causes, such as COPD and cirrhosis^9^,^10^,^11^. In a previous Chinese study, Gu *et al*. identified an increased risk of death among underweight participants (HR = 1.65, 95% CI: 1.54–1.77), which remained consistent after the exclusion of chronic diseases at baseline^12^. Other studies have also revealed an increased risk of death among individuals with low BMI. Both the KCPS (Korean Cancer Prevention Study)^13^ and CPS (Cancer Prevention Study) II studies^7^ showed a similar relative risk for the association between underweight and risk of mortality, and in NHANES^14^, the relative risk for underweight individuals was even higher overall.

Reverse causality maybe a great concern for the interpretation of these results. Subjects with a severe disease such as COPD have greater weight loss over time^15^. In our study, we collected information about chronic diseases (such as cancer, cardiovascular disease and diabetes) at baseline and conducted a sensitivity analysis by excluding persons with preexisting chronic diseases, and we observed similar associations for BMI and risk of all-cause mortality among subjects with no previous chronic diseases. Furthermore, exclusion of the first four years of follow-up which may help control against reverse causality did not alter our results.

Overweight and obesity have long been recognized as a predictor of disease and early death^16^. However, whether overweight truly increases the risk of death is still controversial. We did not find significant excess mortality associated with overweight, which was consistent with a meta-analysis of observational studies that examined the association of all-cause mortality with standard BMI categories as defined by the World Health Organization^8^. This meta-analysis included 97 studies with a combined sample size of 2.88 million and more than 270,000 deaths. The HRs relative to normal weight showed that only obesity was associated with increased mortality (HR = 1.18, 95% CI: 1.12, 1.25); interestingly, overweight was found to be protective for all-cause mortality (HR = 0.94, 95% CI: 0.91, 0.96). Furthermore, when obesity was examined by subcategory, severe obesity was responsible for the increased mortality. They found pooled HRs were 0.92 (95% CI: 0.88–0.96) for overweight among the North American population and 0.96 (95% CI: 0.92–1.00) among the European population. The meta-analysis only included four Chinese studies, and a summary HR for overweight was not available for the Chinese population. On the other hand, a number of large cohort studies have revealed a positive association of overweight with risk of mortality^7^,^12^,^13^,^17^,^18^,^19^,^20^,^21^, including the Nurses’ Health Study^18^, the Physicians Health study^19^, the CPS I^20^, the CPS II^7^, the Korean Cancer Prevention Study^13^, a pooled analysis of Asian Americans^21^ and two Chinese cohort studies^12^,^17^. The discrepancies between the current study and these previous studies could be the referent group used, the different populations, and the confounding factors used for model adjustment. Also, BMI is a proxy for body fat but it does not directly measure body fat or the distribution of body fat, which may prove to be a better predictor of the association between body fat and mortality.

As reported in several epidemiological studies, age was an effect modifier for the relationship between BMI and all-cause mortality^20^,^22^,^23^,^24^. However, most of these studies had inadequate sample size to analyze the effect of a low BMI on all-cause mortality across age groups. In our study, we analyzed by stratifying the age of study participants into two groups (<60 years and ≥60 years), and found evidence of significantly increased risk of mortality among participants aged less than 60 years with BMIs of 15.0–22.4.

As revealed in several studies, smoking was associated with lower body weight and an increased risk of mortality, and can therefore distort the relationship between BMI and risk of mortality^25^,^26^,^27^,^28^, and adjustment for smoking cannot fully address the issue. Sensitivity analysis by excluding smokers is a more powerful method to address this potential bias. Limiting the analyses to nonsmokers slightly attenuated the elevated risk of death among low BMI participants and the decreased risk among persons with high BMI. We also limited the primary analyses to persons who never drank, and the results did not change.

This study has several strengths, including its prospective design and large sample size (including 372,793 persons). We also adjusted for a number of important potential confounders in the multivariate model including age at baseline, sex, smoking, alcohol drinking, education levels, marital status and physical activity. As mentioned previously, we performed sensitivity analyses limiting our evaluation to participants who had never smoked or had no history of chronic disease, and this sensitivity analyses did not alter our findings, so we canbe more certain that low baseline BMI levels in study participants contributed directly to the risk of mortality.

Our study had several limitations. One of the difficulties in examining the relationship between BMI and mortality is the rate of weight change (whether it be loss or gain). In the National Institutes of Health-AARP Diet and Health Study cohort^29^, the changes in weight between ages 18 and 35 years, ages 35 and 50 years, and ages 50 and 69 years were recorded. They demonstrated that weight gain between ages 18 and 35 years or between ages 35 and 50 years was positively associated with risk of mortality. In our study, we only had a single measurement for BMI at baseline, possibly contributing to misclassification of underweight, overweight or obesity status, which may have affected our results. In addition, our study did not collect any information on blood lipids, blood pressure or blood glucose. Dyslipidemia, hypertension and diabetes mellitus have been proved to be positively related to obesity^30^,^31^. These intermediate factors related to the disease may distort the effect of BMI on risk of all-cause mortality. Finally, errors in self-reported height and weight data may have biased the estimates. A meta-analysis has revealed a generally lower summary HR in studies using measured data than in studies using self-reported data^8^, although the correlation between BMI based on these two types of data is typically very high (greater than 0.9)^32^. Thus, bias of the observed association between BMI and risk of mortality due to this type of misclassification is probably minimal.

In summary, we found a relatively increased risk of all-cause mortality among low BMI participants and a relatively lower risk of all-cause mortality among those higher BMI in this YHIS population. Our results did not materially alter after excluding those persons who were smokers, had previous chronic diseases, or were followed up less than four years. Additional studies are needed to confirm these findings, particularly among Chinese populations where there is a different range of BMI than in Western populations.

## Methods

### Study population

Between 2004 and 2009 we enrolled 384,533 Chinese aged 18 years or older in Ningbo, Zhejiang Province, China. Ningbo is a coastal city in the south of the populous Yangtze River Delta, and Yinzhou is the largest District in Ningbo, with a population of nearly 800,000 residents ≥18 years old. In 2004, Yinzhou implemented a high quality Health Information System, which collects all of the residents’ health care records in a timely manner from all hospitals in Ningbo. This Yinzhou Health Information System (YHIS) was the source for the baseline characteristics data for our study population.

Of the study participants, 901 were enrolled in 2004, 266,488 were enrolled in 2005, 31,866 in 2006, 20,023 in 2007, 21,099 in 2008 and 33,204 in 2009. We excluded 10,639 subjects with missing BMI values and 1101 participants with extremely low or high BMI (those lower or higher than the mean BMI ±3 standard deviations). Overall, the final analysis included 372,793 individuals (178,333 men and 194,460 women). This study was approved by the Institutional Review Boards of the Research Center for Air Pollution and Health of Zhejiang University, and all participants gave informed consent for the use of their data. All methods were carried out in accordance with the approved guidelines.

### Data collection

In local village hospitals, data on demographic characteristics, and lifestyle risk factors were obtained using a standard questionnaire administered by trained staff. Most participants had their weight and height measured by trained staff, while a small number of subjects self-reported height and weight. Cigarette smokers (including ever or current smokers) were defined as having smoked at least one cigarette per day for one year or more. Information regarding the amount and type of alcohol consumed by each participant during the past 12 months was also collected. Alcohol consumption was defined as drinking any alcohol during the last year (occasional drinkers: drinking any alcohol less than three days/ week; often drinkers: more than three days/ week). Body weight were measured while subjects were wearing light clothing without shoes, using a standard protocol, and height was measured with the participant standing on a firm, level surface. Body mass index was then calculated as weight in kilograms divided by height in meters squared.

### Mortality follow-up

Vital status was monitored by trained staff from the Yinzhou Center for Disease Control and prevention (CDC), and abstracted from official death certificates. Two CDC staff who were blinded to the study participants’ baseline characteristics independently verified the cause of death, and discrepant results were adjudicated by joint review. Underlying causes of death were coded according the 10^th^ International Classification of Diseases (ICD-10). Vital status was ascertained by linkage of the selected population to the Yinzhou Death Database File in Ningbo, China, with the most recent update on December 31, 2014.

### Statistical analysis

The BMI was divided into ten categories: less than 15.0, 15.0 to 17.4, 17.5 to 19.9, 20.0 to 22.4, 22.5 to 24.9, 25.0 to 27.4, 27.5 to 29.9, 30.0 to 32.4, 32.5 to 34.9, and ≥35.0. Standard categories for BMI (18.5, 18.5–24.9, 25.0–29.9, and ≥30.0) were used in sensitivity analyses. Person-years of follow-up were calculated from the date of the baseline examination until the date of death or December 31, 2014. Age-standardized mortality was calculated using the ten-year age specific mortality and the age distribution of the Chinese population year 2000 census. Cox proportional hazards regression models were used to evaluate the association between the baseline BMI and risk of all-cause mortality. We performed analyses in men and women separately and adjusted for age at baseline (continuous variable), sex(men or women), tobacco smoking (non-smoking, former smoker, or current smoker), alcohol drinking(none, occasional, or often), marital status (married or others), education levels (less than six years or ≥six years) and physical activity (less than one time/week, one to four times/week, or more than four times/week). Crude and adjusted hazard ratios were calculated using participants with a BMI of 22.5–24.9 as a reference group. Stratified analyses were conducted by age at baseline (<60 years or ≥60 years, based on the age distribution in all study participants), sex (men or women), smoking (yes or no), previous chronic disease (yes or no), duration of follow-up (<four years or ≥ four years).

The shape of the association between BMI and mortality was explored using the median of BMI in each category as a continuous variable in the Cox proportional hazards regression models. We also fit continuous models with the addition of a square term to investigate possible non-linear associations. *P* values for the quadratic term from the Cox proportional hazards regression models were higher than 0.05. All statistical analyses were conducted using SAS software (version 9.2, SAS Institute Inc., Cary, NC, USA). All tests were two sided and *P* < 0.05 was considered statistically significant.

## Additional Information

**How to cite this article**: Wang, J.-B. *et al*. Body Mass Index and Mortality: A 10-Year Prospective Study in China. *Sci. Rep.* **6**, 31609; doi: 10.1038/srep31609 (2016).

## Figures and Tables

**Table 1:** Baseline characteristics of the study population according to Body Mass Index.

Characteristics	Body Mass Index^a^										
	<15.0	15.0–17.4	17.5–19.9	20.0–22.4	22.5–24.9	25.0–27.4	27.5–29.9	30.0–32.4	32.5–34.9	≥35.0	*P* values
No. of participants	210	3384	37326	140462	131610	43693	12257	3082	588	181	
Age at baseline (years), median (IQR)	57.0 (41.0,74.0)	53.0 (35.0,68.0)	43.0 (32.0, 58.0)	42.0 (33.0, 54.0)	45.0 (36.0,55.0)	49.0 (40.0,57.0)	52.0 (42.0,59.0)	53.0 (43.0,60.0)	55.0 (46.0,62.0)	54.0 (46.0,62.0)	<0.001^b^
BMI (kg/m^2^), median (IQR)	14.3 (13.7,14.7)	16.9 (16.4,17.3)	19.3 (18.7,19.6)	21.5 (20.8, 22.0)	23.5 (23.0,24.2)	25.9 (25.4,26.6)	28.3 (27.8,29.0)	30.9 (30.4,31.5)	33.3 (32.9,34.1)	36.1 (35.4,37.2)	<0.001^b^
Marital status, No. (%)											
Married	155 (73.8)	2586 (76.4)	29865 (80.0)	118038 (84.0)	114862 (87.3)	38464 (88.0)	10775 (87.9)	2676 (86.8)	491 (83.5)	161 (89.0)	<0.001
Others	55 (26.2)	798 (23.6)	7461 (20.0)	22424 (16.0)	16748 (12.7)	5229 (12.0)	1482 (12.1)	406 (13.2)	97 (16.5)	20 (11.0)	
Education, No. (%)											
Less than 6 years	132 (62.9)	1850 (54.7)	16261 (43.6)	57724 (41.1)	60868 (46.3)	24369 (55.8)	7651 (62.4)	2079 (67.5)	416 (70.8)	133 (73.5)	<0.001
≥ 6 years	78 (37.1)	1534 (45.3)	21065 (56.4)	82738 (58.9)	70742 (53.7)	19324 (44.2)	4606 (37.6)	1003 (32.5)	172 (29.2)	48 (26.5)	
Smoking, No. (%)											
Never	174(84.1)	2728(81.0)	29173(78.7)	104776(75.1)	95084(72.7)	31694(72.8)	8903(73.0)	2361(77.0)	497(84.8)	166(92.2)	<0.001
Former	10(4.8)	187(5.6)	1687(4.5)	6429(4.7)	7012(5.3)	3057(7.0)	1032(8.5)	254(8.3)	32(5.5)	5(2.8)	
Current	23(11.1)	451(13.4)	6229(16.8)	28202(20.2)	28773(22.0)	8763(20.1)	2264(18.5)	450(14.7)	57(9.7)	9(5.0)	
Drinking, No. (%)											
Never	185(89.4)	2942(87.4)	31381(84.6)	112981(81.0)	102451(78.3)	33674(77.4)	9443(77.5)	2511(82.0)	519(88.7)	170(94.5)	<0.001
Occasional	7(3.4)	138(4.1)	1838(5.0)	8588(6.2)	8450(6.5)	2654(6.1)	739(6.0)	134(4.4)	22(3.8)	6(3.3)	
Often	15(7.2)	286(8.5)	3859(10.4)	17842(12.8)	19923(15.2)	7159(16.5)	2008(16.5)	416(13.6)	44(7.5)	4(2.2)	
Physical activity, No. (%)											
Less than 1 time/ week	74(36.8)	1091(33.0)	10738(29.2)	38026(27.4)	34692(26.7)	11128(25.7)	3082(25.5)	753(24.8)	142(24.4)	38(21.5)	<0.001
1 to 4 times/ week	72(35.8)	1122(33.9)	11989(32.6)	43515(31.4)	42735(32.8)	15463(35.8)	4276(35.4)	1113(36.7)	217(37.3)	65(36.7)	
More than 4 times/ week	55(27.4)	1092(33.1)	14002(38.1)	57076(41.2)	52669(40.5)	16630(38.5)	4725(39.1)	1169(38.5)	223(38.3)	74(41.8)	

Abbreviations: BMI = body mass index; IQR = interquatile range.

^a^Body mass index was calculated as weight in kilograms divided by the square of height in meters.

^b^The Kruskal-Wallis Test.

**Table 2:** Crude and age-adjusted mortality rate according to BMI categories.

BMI	N	Deaths	Person-years	Crude mortality rate (per 10^5^ person-years)	Age-adjusted mortality rate* (per 10^5^ person-years)
<15.0	210	40	1721.5	2323.6	828.5
15.0–17.4	3384	297	27695.2	1072.4	459.4
17.5–19.9	37326	1885	310528.27	607.0	385.5
20.0–22.4	140462	4968	1180809.2	420.7	381.6
22.5–24.9	131610	4073	1106778.4	368.0	359.0
25.0–27.4	43693	1151	363821.6	316.4	275.5
27.5–29.9	12257	332	101904.7	325.8	239.4
30.0–32.4	3082	74	25589.6	289.2	212.9
32.5–34.9	588	15	4901.7	306.0	276.1
≥35.0	181	8	1493.6	535.6	261.7

^*^Age-adjusted mortality was calculated using the 10-year age specific mortality and the age distribution of the Chinese population 2000 year census data.

BMI: Body Mass Index.

**Table 3:** Hazard ratios (HRs) and 95% confidence intervals (95% CIs) for all-cause mortality, according to Body Mass Index.

Variables	Body Mass Index									
	<15.0	15.0–17.4	17.5–19.9	20.0–22.4	22.5–24.9	25.0–27.4	27.5–29.9	30.0–32.4	32.5–34.9	≥35.0
All subjects	210	3384	37326	140462	131610	43693	12257	3082	588	181
No. of deaths	40	297	1885	4968	4073	1151	332	74	15	8
Age and sex adjusted HR (95% CI)	1.73 (1.26–2.36)	1.11 (0.99–1.25)	1.05 (0.99–1.11)	1.06 (1.01–1.10)	1.00	0.73 (0.68–0.78)	0.66 (0.59–0.74)	0.56 (0.44–0.70)	0.46 (0.28–0.76)	0.99 (0.49–1.97)
Multivariate HR^a^ (95% CI)	1.61 (1.17–2.23)	1.07 (0.94–1.20)	1.04 (0.98–1.10)	1.06 (1.02–1.11)	1.00	0.73 (0.68–0.78)	0.66 (0.58–0.74)	0.53 (0.42–0.68)	0.48 (0.29–0.80)	0.77 (0.35–1.71)
All men										
No. of deaths	16	157	945	2693	2194	514	133	23	3	0
Multivariate HR^b^ (95% CI)	2.01 (1.23–3.29)	1.28 (1.08–1.51)	1.11 (1.02–1.20)	1.07 (1.01–1.14)	1.00	0.68 (0.61–0.75)	0.59 (0.49–0.71)	0.46 (0.30–0.71)	0.45 (0.15–1.42)	-
All women										
No. of deaths	24	140	940	2275	1879	637	199	51	12	8
Multivariate HR^b^ (95% CI)	1.38 (0.90–2.12)	0.89 (0.75–1.07)	0.96 (0.89–1.04)	1.04 (0.98–1.11)	1.00	0.78 (0.71–0.86)	0.72 (0.62–0.83)	0.58 (0.43–0.78)	0.49 (0.28–0.87)	0.87 (0.39–1.94)
<60 y of age										
No. of deaths	3	41	300	1232	1175	316	81	19	1	1
Multivariate HR^a^ (95% CI)	1.79 (0.45–7.16)	2.10 (1.52–2.88)	1.19 (1.05–1.36)	1.15 (1.06–1.25)	1.00	0.73 (0.65–0.83)	0.66 (0.52–0.83)	0.60 (0.37–0.97)	0.20 (0.03–1.43)	0.75 (0.11–5.33)
≥60 y of age										
No. of deaths	37	256	1585	3736	2898	835	251	55	14	7
Multivariate HR^a^ (95% CI)	1.51 (1.08–2.11)	0.96 (0.84–1.09)	0.98 (0.92–1.05)	1.02 (0.97–1.07)	1.00	0.74 (0.68–0.80)	0.67 (0.59–0.77)	0.53 (0.40–0.70)	0.54 (0.32–0.92)	0.78 (0.32–1.87)

^a^Adjuested for age at baseline, sex, smoking, drinking, education level, marrital status and physical activity.

^b^Adjusted for age at baseline, smoking, drinking, education level, marrital status and physical activity.
